# Health Information Technologies in a Resource-Limited Setting: Knowledge, Attitude, and Practice of Health Professionals

**DOI:** 10.1155/2023/4980391

**Published:** 2023-02-01

**Authors:** Nebyu Demeke Mengestie, Abraham Yeneneh, Asefa Birara Baymot, Mulugeta Hayelom Kalayou, Mequannent Sharew Melaku, Habtamu Alganeh Guadie, Genet Paulos, Wondwossen Zemene Mewosha, Aynadis Worku Shimie, Ashenafi Fentahun, Sisay Maru Wubante, Masresha Derese Tegegne, Shekur Mohammed Awol

**Affiliations:** ^1^Department of Health Informatics, Institute of Public Health, University of Gondar, Ethiopia; ^2^Meket Woreda Health Office, North Wollo Zone, Amhara Region, Ethiopia; ^3^Department of Health Informatics, Institute of Public Health, Wollo University, Ethiopia; ^4^Institute of Public Health, Bahir Dar University, Ethiopia; ^5^International Institute for Primary Healthcare, Ethiopia

## Abstract

The use of health information technology significantly enhances patient outcomes. As a result, policymakers from developing countries have placed strong emphasis on formulating eHealth policies and initiatives. However, there have not been many successful deployments to show for. The role of individual factors in the successful implementation of these technologies is indispensable. Therefore, this study assesses healthcare professionals' knowledge, attitudes, and practice of health information technology. An institution-based cross-sectional study was conducted at the University of Gondar Comprehensive Specialized Hospital from November 15 to December 29, 2020. A structured, self-administered questionnaire was used to collect data. Student's *t*-test was used to learn if there were any significant differences in practice habits between participants with and without previous information technology-related training. In addition, first-order partial correlation was conducted to identify the relationship of knowledge and attitude with practice. A total of 347 health professionals responded to the questionnaire, yielding an 87.2% response rate. Most health professionals are not aware of how to use health information technologies. Notably, practice levels were low and needed prompt action from responsible authorities. Previous training did not work very well to improve the practice levels of health professionals. However, the positive attitude of these professionals encourages policymakers and implementers to engage closely.

## 1. Introduction

Health information technology (HIT) is the application of information processing involving both computer hardware and software that deals with the storage, retrieval, sharing, and use of healthcare information, data, and knowledge for communication and decision making [1]. This definition dates to 2004. Nonetheless, it still serves as a basis for defining the concept of HIT and related works have used this definition continually [2, 3]. For instance, the term computer in today's technology lead world implies a variety of electronic devices that are capable of processing information. The definition is a cornerstone to the ever-evolving ecosystem of information technology.

The advancement in technology has brought significant transformations in the health sector [4, 5]. These advancements have made healthcare services accessible [6], affordable [7], patient centered [4, 8], less prone to error [5, 9], and interactive [10]. Despite continuous efforts, the adoption of technologies in developing countries like Ethiopia has proven to be intricate [11]. This low adoption stems from a variety of origins [11–13]. Organizational establishments and user-related factors take the lion share in the factor list. Organizational establishments like service quality, system quality, and training are at the top of the queue [11, 14]. Equally important are individual factors like user behaviors, practice habits, and level of knowledge [15]. Users of technologies in the healthcare arena can dictate the success and failure of health information technologies [12, 16]. Different studies have underscored how crucial a user-centered approach is to implementing HITs [17, 18]. However, such approaches usually focus on developing a technology perceived to be suitable for the user albeit overlooking the users' peculiar background. That is why we need to assess users' background in terms of knowledge, attitudes, and practice of such technologies. This would inform how much work is needed before thinking about implementing or even developing eHealth systems in the health sector. Healthcare professionals who deliver health services are indispensable in the utilization and implementation of HITs to enhance health service delivery. Developing countries like Kenya, Ethiopia, Mali, and others have formulated policies that direct towards the technological transformation of the health sector [19, 20]. Healthcare professionals take key roles in these policies. In Ethiopia, the road towards health information revolution began in 2016 and it involves capacity building of healthcare professionals [19]. Thus, healthcare professionals are expected to possess knowledge, attitude, and practice of HITs. This study will assess the level of knowledge, attitude, and practice of HITs.

## 2. Method

### 2.1. Aim, Design, and Setting of the Study

An institution-based cross-sectional study was conducted to determine the knowledge, attitude, and practice of health information technology among health professionals at the University of Gondar (UoG) Comprehensive Specialized Hospital from November 15 to December 29, 2020. The UoG Comprehensive Specialized Hospital serves a population of around 4 million. On average, 1300 patients visit the hospital daily. At the time of data collection, the hospital had around 1004 staff members distributed across 30 departments.

### 2.2. Sampling and Participants

**Figure 1:**
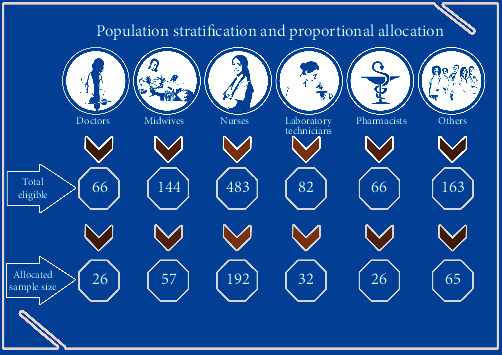
Sampling procedure and stratification of health professionals at University of Gondar Comprehensive Specialized Hospital 2020.

Participants of this study are all health professionals working at the UoG Comprehensive Specialized Hospital. Sample size was determined using the single population proportion formula. By taking a 95% confidence, 5% margin of error, and 50% of proportion, the sample size was determined to be 398. Before drawing samples from the study population, health professionals were stratified based on their respective professions to ensure the representativeness of the sample. Medical doctors, nurses, laboratory technicians, midwives, pharmacists, and others were included as a stratum in the process of drawing samples. Then, participants were selected randomly from a sampling frame containing all eligible health professionals according to the identified strata. Detailed information on the sampling can be found in Figure 1.

### 2.3. Measurement and Operational Definitions

*Health information technology*: it is defined as the use of electronic devices by health professionals for health promotion, prevention, treatment, and rehabilitation purposes.

*Knowledge*: knowledge as a single variable is difficult to measure; thus, it was classified in three dimensions [21]. The three dimensions of knowledge in health information technology and their respective measurements are as follows: knowing the definition of HIT was assessed by 10 items, knowing its area of application was assessed by 6 items, and knowing the methods of utilization was assessed by 6 items. All the knowledge items have “yes” and “no” response options. Taking Bloom's classification as a base, participants who scored ≥80% were labeled as having “good” knowledge, participants who scored 60%-79% were labeled as having “intermediate” knowledge, and participants who scored less than 60% were labeled as having “poor” knowledge [22].

Attitude was measured using an eight-item scale with a 5-point Likert response [21]. Similarly, practice was measured using an eight-item scale with a 5-point Likert response indicating level of use [21]. Responses for the Likert scale questions are valued from “1” up to “5” making the maximum possible sum of values 40 and the minimum 8 for both variables. Categorizing of attitude and practice was done using the demarcation threshold formula to decrease subjectivity [23]. A score of greater than 29 indicates a favorable attitude, and a score of greater than 27 indicates a high level of practice.

A pretest was administered to 5% of the total sample size. The participants of the pretest completed the questionnaire and then forwarded their feedback on the questions. The final questionnaire was then modified following the analysis of the pretest results and feedback. Internal consistency of the attitude scale and the practice scale was checked using Cronbach's alpha, resulting in scores of 0.84 and 0.78, respectively. Internal consistency of the knowledge scale was checked using the Kuder-Richardson-20 method, resulting in a score of 0.80 across the three dimensions.

### 2.4. Statistical Analysis

Data entry was conducted using Epi Info 7.2 to minimize errors during the entry process. Data analysis was performed by the Statistical Package for the Social Sciences (SPSS) version 25. Descriptive statistics for both independent and dependent variables were computed. A *t*-test was also conducted to determine if there is a significant difference in HIT practice between those who took information communication technology training and those who did not. The normality of the practice scores was checked using the Shapiro-Wilk test. The normality assumption was met with a *P* value of 0.34. A *P* value of less than 0.05 and a confidence interval of 95% were used for interpretation. The relationship between knowledge, attitude, and practice was assessed with first-order partial correlation, and the Pearson correlation coefficient was used to describe the association.

## 3. Results

### 3.1. Participant Characteristics

A total of 347 health professionals responded to the questionnaire, yielding 87.2% response rate. Majority of the participants were male 202 (58.2%) and about 227 (65.4%) were less than 30 years of age. Profession-wise, nurses 166 (47.8%) were predominant in the study. The largest group, experience-wise, was composed of participants with five up to nine years of experience 130 (37.5%), and educational level-wise, the majority was composed of participants with undergraduate degree 217 (62.5%). Regarding training, majority of the participants 249 (72%) had taken information technology-related training(s) in the past five years. Only half 172 (49.6) of the participants reported that they got continuous support from staff members regarding the use of information technology tools.  Table 1 shows detailed information about the participants in this study.

### 3.2. Knowledge

Knowledge was classified and described in three dimensions. The first one is knowing the definition of HIT, and more than half 204 (58.8%) of the participants had good knowledge regarding the definition of HIT. The second dimension is knowledge on the methods of using HIT, and only 49 (14.1%) of the participants had good knowledge. Majority of the participants 281 (81%) had intermediate-level knowledge regarding the methods of using HIT. Notably, only 17 (4.9%) of the participants had poor knowledge on this dimension. The third and final dimension is knowledge on the application areas of HIT, and almost one-third 103 (29.7%) of the participants had a good knowledge concerning this dimension. Overall, only 20 (5.8%) of the participants had good knowledge on all three dimensions of knowledge on HITs. However, 82 (23.6%) of the participants had a good knowledge on at least two dimensions of knowledge about HITs.

### 3.3. Attitude

Attitude was measured using eight items having 5-point Likert responses each. Eight was the minimum score, and 40 was the maximum score on the attitude scale. The median attitude score was 28 with an interquartile range of 5. Two-thirds 230 (66.3%) of the participants had a favorable attitude towards HITs. The majority were male, 136 (39.2%); greater than or equal to 28 years of age, 122 (35.2%); nurses, 117 (33.7%); holders of bachelor's degree, 143 (41.2%); and married, 116 (33.4%). Regarding trainings, from the 249 participants who took information technology training(s), 173 (69.5%) of them have a favorable attitude towards HITs. Detailed information can be found in Table 2.

### 3.4. Practice

**Figure 2:**
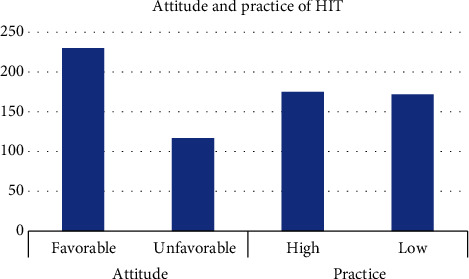
Attitude and practice of health professionals at University of Gondar Comprehensive Specialized Hospital 2020.

Level of practice of HITs was measured using an eight-item scale with a 5-point Likert response. The mean practice score was 23.5 (standard deviation ± 7.6). Only half 175 (50.4%) (45.0%-55.3%) of the participants had a high level of HIT practice. Normality was checked using the Shapiro-Wilk test and resulted a *P* value of 0.07, which confirms a normal distribution. In a like manner, visual inspection of the graphs illustrated the same result. From the total of 175 participants with a high level of HIT practice, the majority 110 (62.8%) were male, 101 (57.7%) were greater than or equal to 28 years of age, 108 (61.7%) were supported by their staff, 128 (73.1%) had taken training(s), 95 (54.2%) were married, and regarding professions of the participants, 82 (46.8%) were nurses. Detailed description of the practice levels is presented in Table 2.  Figure 2 shows a comparison of both attitude and practice levels.

### 3.5. Training and Practice

An independent sample *T*-test was conducted to compare if a significant difference exists regarding the practice of HIT between health professionals who took information technology training in the past five years and those who did not. There was no significant difference in the HIT practice scores between those who had taken training(s) (*M* = 23.7, SD = 7.6) and who did not (*M* = 23.1, SD = 7.6); *t*(345) = 0.69, *P* = 0.261. Therefore, these results suggest that the training(s) the participants have taken did not impact their practice of HITs.

### 3.6. Correlation of Knowledge and Attitude with Practice

First-order partial correlation was performed to assess the relationship between practice and knowledge and practice with attitude. The finding shows that by controlling for the effect of knowledge on attitude and practice, it was found that attitude and practice have a positive significant association, *r* = 0.39, *p* (two-tailed) < 0.001. In contrast, by controlling for the effect of attitude on knowledge and practice, it was found that practice and knowledge were not significantly associated.  Table 3 shows detailed information about the partial correlation conducted.

## 4. Discussion

This study uncovered that the knowledge, attitude, and practice of health information technology among healthcare professionals range from low to moderate. In addition, this study found that ICT-related training taken by the participants did not significantly affect the practice habits of health professionals.

A 2020 study in an Egyptian hospital among 205 nurses found that 97.1% of them had good level of knowledge [24]. This finding is higher than the current study's finding of a 23.6% level of good knowledge. The difference might be because the former study is conducted among health professionals who are working in a hospital where there is an established network and electronic health information system [24]. The more exposed a health professional is to electronic systems, the better the level of knowledge. A 2019 study among doctors working in a tertiary health facility in Bangladesh found that 62% had a good level of knowledge which is higher than the current study's findings. In addition to differences in settings, this difference might be caused by an overestimation due to the unscientific reduction of sample size in the Bangladeshi study [25].

A 2018 study in Ethiopia among health professionals in three public health facilities found that 37.6% had a good level of knowledge about telemedicine which is one of the applications of HITs [26]. This finding is higher than the current study's finding. Since those who know telemedicine well might not necessarily know the whole of HITs', there might be a gap in the overall knowledge levels. Similarly, a 2020 study in the Plateau State of Nigeria found that 71.5% of health professionals who worked at the frontline had good level of knowledge [27]. This is supported by a 2016 study among health professionals working at teaching hospitals in Puducherry Union Territory, India, which found that 41% had good level of knowledge which is higher than the current study's finding [28].

Concerning knowledge about HITs, a 2017 study in an Iranian hospital among healthcare activists found that only 28.8% of the participants had good knowledge, while in the current study, 23.6% of the participants have good knowledge [29]. A 2020 study among doctors who work in public health facilities in Pakistan found that 80.7% of the participants had good level of knowledge about the definition which is higher than the current study's finding. However, the overall level of knowledge agreed with the current study [30]. Another study among healthcare professionals and medical students at five teaching referral hospitals in Teheran, Iran, found that a total of 28.1% respondents had a good level of knowledge which is higher compared to the current study's 5.8% [31].

A similarly structured study in 2016 among 112 medical doctors in Bangladesh private hospitals found that 50% had average knowledge of eHealth and about 26% had good knowledge, whereas in the current study, only 5.2% had a good level of knowledge in all knowledge dimensions. Regarding attitude the Bangladeshi study among doctors found 78% had a favorable attitude which is higher compared to the current study's finding of 66.3% [15].

Nonetheless, a 2017 study on healthcare providers from 6 Kuwaiti hospitals found that 41.6% of the providers had low level of knowledge in knowing the definition of HIT, while the current study's 58.8% result underlines a significant difference. Similarly, regarding the domain of knowing method of utilizing HIT, the current study found that only 14.1% of the participants had good level of knowledge, while the Kuwaiti study found 22.6%, which signifies a slight increase [21].

Regarding attitude and practice, the Iranian study among healthcare activists measured both together and found the percentage of good attitudes and practices to be 40%, compared to the current study's finding of attitudes and practices of 66.3% and 50.4%, respectively [29]. The time difference in the two studies might have affected healthcare professionals' knowledge and attitudes as recent developments in technology are likely to bring positive changes.

A 2018 study among doctors working at the Lagos University Teaching Hospital, Nigeria, found that all the participants had a favorable attitude towards HITs [32]. The current study found that 66.3% of the participants had a favorable attitude. The Nigerian study is conducted during a postimplementation phase of an electronic medical record system. Therefore, the quality of the system might have influenced the attitudes of the participants [32]. Similarly, a 2020 study among Dutch general practitioners (GPs) found that majority had a positive attitude towards eHealth and online diagnostic testing which are applications of HITs [33]. The observed difference between the current study might be due to the Dutch GPs' universal use of eHealth systems which they evaluated as efficient and smoothly running system. The Bangladeshi study among doctors found that 59% of them possessed a favorable attitude towards HITs which agrees with findings of the current study [25]. These findings are also in agreement with the study from Pakistan which found more than average favorable attitude and the study from Ethiopia [26, 30, 34]. In contrast, the study from India found that only 39% of the participants had favorable attitude which is lower as compared to 66.3% finding of the current study [28].

Regarding practice of HITs, a 2022 study among health professionals from rural areas of Sindh in Pakistan found that 52.5% practiced telemedicine which is a similar finding to the current study [35]. The study from India found that 44% of the health professionals practice well which is lower than the current study's finding of 56% [28]. In addition, the study from Iran found that 40.4% had good attitude and practice habits; however, the current study found 66.3% and 50.4% attitude and practice levels, respectively [31]. These disparities might be attributed to the varying levels of digitalization in the workplaces of healthcare professionals [36]. The study from Sindh, Pakistan, found that taking training positively influences practice levels of health professionals [35]. However, in the current study, it was found that taking training was not related to practice habits of health professionals. This difference underlines that focus shall be given not only on delivering trainings but also on tailoring high-quality trainings.

## 5. Conclusion

Hitherto, developing countries like Ethiopia have struggled to digitize the health sector. Knowledge, attitudes, and practice of health information technology are essential elements that pave the way towards digitizing the health sector. In this study, knowledge about the definition of HIT is more prevalent, while knowledge about the method of use and application areas of health information technology is lower. Health information technology practice was moderate. Previous information technology-related training delivered to health professionals did not significantly impact the practice of health information technology. Attitude of healthcare professionals towards HIT was high, handing more positive prospects to future implementations. Further research is required to determine why trainings fail to produce the desired results.

The higher level of attitude shows that future digital interventions in the health sector might be welcomed by health professionals although the manner of delivering these interventions needs scrutiny from the standpoint of policymakers and implementers.

## Figures and Tables

**Table 1:** Sociodemographic characteristics of health professionals at University of Gondar Comprehensive Specialized Hospital, 2020.

Characteristic	Frequency (%)
Gender	
Male	202 (58.2%)
Female	145 (41.8%)
Age groups (years)	
<30	227 (65.4%)
30-39	105 (30.3%)
40-49	9 (2.6%)
>50	6 (1.7%)
Professions	
Nurse	166 (47.8%)
Laboratory	29 (8.4%)
Doctor	25 (7.2%)
Pharmacy	22 (6.3%)
Midwife	50 (14.4%)
Others	60 (15.9%)
Experience	
<5	112 (32.2%)
5-9	130 (37.5%)
≥10	105 (30.2%)
Training(s)	
Yes	249 (71.8%)
No	98 (28.2%)
Get continuous support	
Yes	172 (49.6%)
No	175 (50.4%)
Education level	
Diploma	78 (22.5%)
Bachelor's degree	217 (62.5%)
Master's degree	25 (7.2%)
Medical doctor	25 (7.2%)
Specialist	2 (0.6%)

**Table 2:** Attitude and practice of health professionals disaggregated by variables at University of Gondar Comprehensive Specialized Hospital, 2020.

Variables	Attitude towards HIT		Practice	
	Favorable	Unfavorable	High	Low
Gender				
Male	136 (39.2%)	66 (19.0%)	110 (37.7%)	92 (26.5%)
Female	94 (27.1%)	51 (14.7%)	65 (18.7%)	80 (23.1%)
Age				
≥28	122 (35.2%)	61 (17.6%)	101 (29.1%)	82 (23.6%)
<28	108 (31.1%)	56 (16.1%)	74 (21.3%)	90 (25.9%)
Profession				
Nurse	117 (33.7%)	49 (14.1%)	82 (23.6%)	84 (24.2%)
Laboratory technician	18 (5.2%)	11 (2.2%)	20 (5.8%)	9 (2.6%)
Medical doctor	19 (5/5%)	6 (1.7%)	5 (1.4%)	20 (5.8%)
Pharmacist	18 (5.2%)	4 (1.2%)	15 (4.3%)	7 (2.0%)
Midwife	22 (6.3%)	28 (8.1%)	25 (7.2%)	25 (7.2%)
Others	36 (10.4%)	19 (5.5%)	28 (8.1%)	27 (7.8%)
Marital status				
Single	101 (29.1%)	60 (18.2%)	70 (20.2%)	91 (26.2%)
Married	116 (33.4%)	48 (14.4%)	95 (27.3%)	69 (20.0)
Divorced	6 (1.7%)	5 (1.4%)	8 (2.3%)	3 (0.9%)
Widowed	6 (1.7%)	5 (1.4%)	7 (2.0%)	4 (1.1%)
Educational level				
Diploma	54 (15.6%)	24 (6.9%)	46 (13.3%)	32 (9.2%)
Bachelor's degree	143 (41.2%)	74 (21.3%)	109 (31.4%)	108 (31.1%)
Master's degree	14 (4.0%)	11 (3.2%)	13 (3.7%)	12 (3.5%)
Medical doctor	19 (5.5%)	6 (1.7%)	7 (2.0%)	18 (5.2%)
Specialist	—	2 (0.6%)	—	2 (0.6%)
Training taken				
Yes	173 (49.9%)	76 (21.9%)	128 (36.9%)	121 (34.9%)
No	57 (16.4%)	41 (11.8%)	47 (13.5%)	51 (14.7%)
Get support from staff				
Yes	124 (35.7%)	48 (13.8%)	108 (31.1%)	64 (18.4%)
No	106 (30.5%)	69 (19.9%)	67 (19.3%)	108 (31.1%)

**Table 3:** Correlation of knowledge and attitude of health professionals with practice of HITs.

Variables	Practice
Knowledge	
*P* value	0.16
*R*	0.25
*R* adjusted	0.18
Attitude	
*P* value	0.00^∗^
*R*	0.48
*R* adjusted	0.39

^∗^ *P* value < 0.001.

## Data Availability

On request, the corresponding author will provide the data that back up the study's conclusions.
